# OCCUPATIONAL EYE LENS DOSE OVER SIX YEARS IN THE STAFF OF A US HIGH-VOLUME CANCER CENTER

**DOI:** 10.1093/rpd/ncaa187

**Published:** 2020-12-15

**Authors:** M B Bellamy, D Miodownik, B Quinn, L Dauer

**Affiliations:** Department of Medical Physics, Memorial Sloan Kettering Cancer Center, New York, NY, USA; Department of Medical Physics, Memorial Sloan Kettering Cancer Center, New York, NY, USA; Department of Medical Physics, Memorial Sloan Kettering Cancer Center, New York, NY, USA; Department of Medical Physics, Memorial Sloan Kettering Cancer Center, New York, NY, USA

## Abstract

This paper summarizes the dose to the eye lens of workers of Memorial Sloan Kettering Cancer Center, a high-volume US oncologic and associated diseases facility. The doses presented in this report were collected from personal dosemeter readings using optically stimulated luminescence badges to estimate H_p_(3). Doses were collected for 5950 clinical and research workers between January 2012 and December 2017. The median eye lens dose for all monitored workers was 0.23 mSv y^−1^. Workers performing, or supporting, fluoroscopy procedures received the highest unprotected eye lens dose of all workers with a median eye dose of 10 mSv. The use of leaded glasses by this group reduced the actual doses to the lens. Nurses and technicians involved in positron emission tomography injections received median eye lens dose of 1.2 mSv.

## INTRODUCTION

The link between ionizing radiation exposure and the formation of lens opacities is well recognized^(1–3)^. Dose limits to the whole body, and to the eye lens, have recently changed because the eye lens has now been recognized as one of the more radiosensitive tissues in the body^(4–8)^. Because vision impairing cataracts and lens opacities lead to the loss of eye lens function, occupational radiation protection programmes should endeavor to avoid, or minimize, this effect in workers^(3)^.

The objective of this paper is to publish lens of the eye doses of a cohort of medical workers in the context of potential updates to regulatory guidance. The US Nuclear Regulatory Commission (NRC) current annual occupational dose limit to the eye lens has been set to 150 mSv^(9)^. However, the International Commission of Radiological Protection (ICRP) has recently recommended a dose limit to the eye lens of 20 mSv y^−1^, averaged over 5 years, with no single year above 50 mSv. Similarly, the National Council on Radiation Protection and Measurements has now recommended a dose limit to the eye lens of 50 mGy y^−1(^^10^^)^. Updates to regulatory limits may have an impact on radiation protection policies of medical institutions.

As a cohort, medical workers have been identified as the largest group of individuals exposed occupationally to ionizing radiation^(11,12)^. There are new implications for medical workers, which have resulted from the understanding of low-dose cataract formation and from the recommended reductions to annual dose limits^(13–15)^. Medical workers involved in certain disciplines, such as interventional cardiology and radiology, have often received annual eye doses that are close to regulatory dose limits^(^^16–20^^)^.

There is growing interest in understanding the health effects associated with low-dose chronic exposures such as those in the medical industry^(21)^. Memorial Sloan Kettering (MSK) is one of the largest cancer centers in the world in terms of patient volume and utilizes a large variety of radionuclides and radiation sources due to the breadth of its cyclotron radionuclide production and diagnostic, therapeutic and research activities. In addition, MSK employs dosimetry for a large worker cohort and has dosimetry records for its workers from as early as 1946. Quantified eye lens exposures for this large medical worker cohort provides context for potential revisions to the dose limits and may help to inform future radiation epidemiology studies.

## METHOD

The eye lens doses presented in this work were based on personal dose monitoring data for occupationally exposed workers associated with MSK. All employees who use, or routinely encounter, radioactive materials or radiation-producing equipment were included in this study. Conversely, MSK employees who did not use or have little potential for radiation exposure were not included in this study. Monitoring badges were issued to employees with potential exposures of 10% or greater of the annual dose limit for radiation. However, over 99% of monitored workers received substantially <10% of the limit due to prolific monitoring. In addition, the doses discussed in this work did not consider the dose reduction effect of any personal shielding devices such as leaded glasses.

Every worker in the study was assigned personnel monitors, which were used to assess doses to the lens of the eye received from radiation sources in the workplace. Workers were instructed that the primary monitor was to be worn on the front of the body above waist level, or in the area of the body with the highest anticipated radiation level. Monitors were issued and collected monthly, or bimonthly, depending on the worker’s assigned section or department. The doses reported in this study were derived from personal monitoring over 6 years from 2012 to 2017.

Doses are presented for variety of categories where medical workers are occupationally exposed to ionizing radiation. These categories are presented in Table 1 and include interventional radiology (IR) and fluoroscopically guided interventional (FGI) doctors and fellows, cyclotron staff, radiopharmacy workers, nuclear medicine technicians and nurses, clinical radiochemistry, medical health physicists, IR and FGI nurses and technicians, radiochemistry researchers, medical physicists, outpatient nurses, radiology staff (non-IR) and inpatient nurses. The exposure details associated with these medical staff categories have been published elsewhere^(22–25)^.

**Table 1:** Average, 25th, 50th and 75th percentiles and maximum annual dose to the eye lens, H_p_(3), broken down by medical staff category. Doses do not consider personal shielding devices

**Category**	**Annual radiation dose to eye lens H**_**p**_ **(3) (mSv y** ^**−1**^ **)**					
	**Average annual LDE** ^a^	**Average annual LDE** ^b^	**Maximum LDE**	**25th percentile** ^b^	**Median LDE** ^b^	**75th percentile** ^b^
IR and FGI doctors/fellows	11	11	45	4	10	16
Cyclotron operations	4.3	4.4	21	1.2	2.8	4.4
Radiopharmacy	2.3	2.6	9.1	0.50	2.0	4.0
Nuclear medicine technicians/nurses	1.3	2.2	20	0.30	1.2	3.0
Clinical radiochemistry	0.79	1.1	4.7	0.21	0.81	1.5
Medical health physics	0.52	0.78	5.2	0.10	0.55	1.0
IR and FGI nurses and technicians	0.50	1.4	25	0.08	0.41	1.8
**All workers**	**0.29**	**1.6**	**45**	**0.06**	**0.23**	**1.3**
Research radiochemistry	0.05	0.56	8.3	0.07	0.14	0.40
Medical physics	0.03	0.22	2.3	0.02	0.10	0.20
Outpatient nurse	0.03	0.27	2.0	0.04	0.10	0.30
Radiology (non-IR)	0.02	0.19	3.6	0.02	0.04	0.16
Inpatient nurse	0.02	0.26	2.0	0.05	0.20	0.30

^a^All monitored workers.

^b^Measurably exposed workers (the subset of workers with doses >0.1 mSv).

Bold values are associated with the entire population of workers.

Although workers were generally assigned a single personnel monitor, two monitors were assigned to persons associated with the Department of Radiology. Collar badges are provided for these workers because they are exposed to inhomogeneous radiation fields due to their routine use of lead aprons. These workers performed angiography or other fluoroscopic procedures. One badge was worn on the collar outside the protective apron, whereas the other, coded whole-body badge, was worn under the apron. For these workers, only the collar badge was used to calculate dose to the eye lens.

The collected badges were read by a US-based commercial dosimetry vendor, Landauer^®^. This vendor is certified by National Voluntary Laboratory Accreditation Program (NVLAP) and uses accredited Luxel+ optically stimulated luminescence (OSL) badges^(26)^ for calculating H_p_(0.07) and H_p_ (9). Calibration of the personnel monitors followed NVLAP requirements^(27)^. The badge’s active volume consists of a thin strip of aluminum oxide (Al_2_O_3_:C) crystal as the active detector immediately surrounded with a multi-element filter pack. This detector recorded the dose associated with X-ray, gamma radiation from 0.01 mSv to 10 Sv and beta radiation from 0.10 mSv to 10 Sv. Regarding detector uncertainty, the 95% confidence interval of H_p_ (3) was ±15% for photons above 20 keV and beta particles above 200 keV.

The eye lens dose was calculated based on personal dose equivalent measurements as outlined in ICRP Publication 103^(1)^. Here, the eye lens dose is defined as dose at a depth of 300 mg cm^−3^ (0.3 cm)^(28,29)^. A regulatory method for calculating eye lens dose was not yet defined in US federal law and is an area of ongoing development^(30–33)^. Thus, under specific irradiation conditions, H_p_(0.07), or H_p_ (9), may be used as a surrogate for H_p_ (3). Univariate statistical analyses were performed in Tableau 9.0, and annual doses to the eye lens were calculated by summing the worker’s monthly, or quarterly, doses. The eye lens doses reported in this work were estimated from H_p_(0.07) and from H_p_ (9) using the following formula by US Landauer^®^ Dosimeters & Radiation Measurement Services based on the International Atomic Energy Agency Technical Documents 1731^(34)^ framework:

$$ {\mathrm{H}}_{\mathrm{p}}(3)={\mathrm{H}}_{\mathrm{p}}(0.07)\times \Big[1.4-\left[1.04\times{\mathrm{e}}^{\left(-\frac{\mathrm{H}\mathrm{p}(10)}{\mathrm{H}\mathrm{p}(0.07)}\right)}\right]\Big] $$

Control dosemeter were stored in an area that only experienced background radiation and were submitted to the badging contractor along with the working badges. The reported dose was calculated as the difference between the dose to worker dosemeter and the dose to the control badge.

## RESULTS AND DISCUSSION

Annual dose to the eye lens for 2012–17 were collected for 5950 unique workers with collective dose^(35)^, mean and standard deviation of 3.1 person-Sv, 0.29 mSv and 1.7 mSv, respectively. Of the workers, 1721 received a measurable dose in at least 1 year of the study duration. The doses ranged from 0 to 45 mSv. The annual collective dose increased by 103% over the period of this study from 0.38 person-Sv in 2012 to 0.77 person-Sv in 2017. The increase in collective dose was driven primarily by nuclear medicine and fluoroscopy workers.

**Figure 1:**
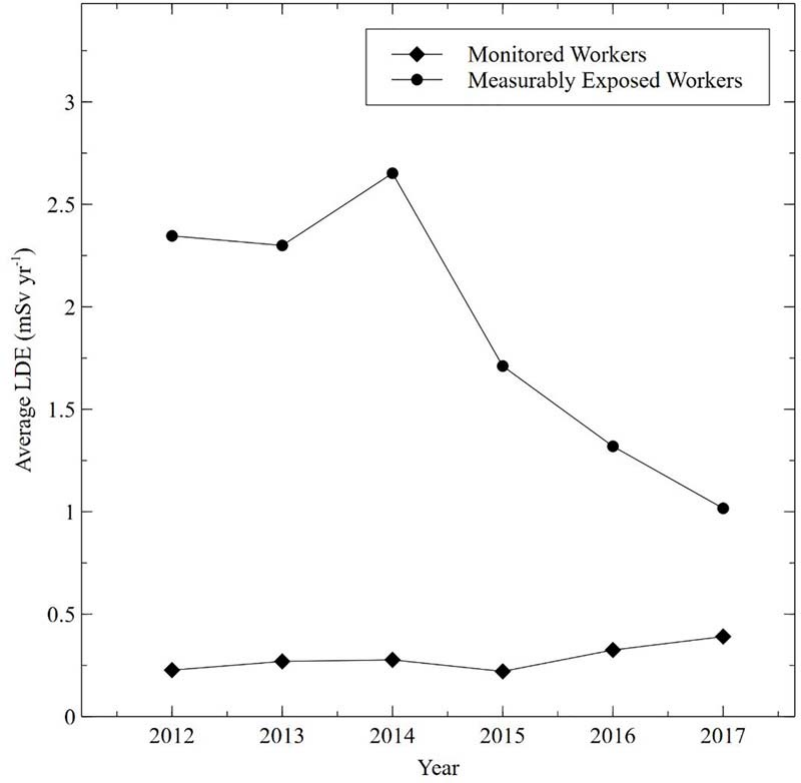
Comparison of the average lens dose equivalent (LDE) (mSv y^−1^) for monitored workers versus measurably exposed workers.

**Figure 2:**
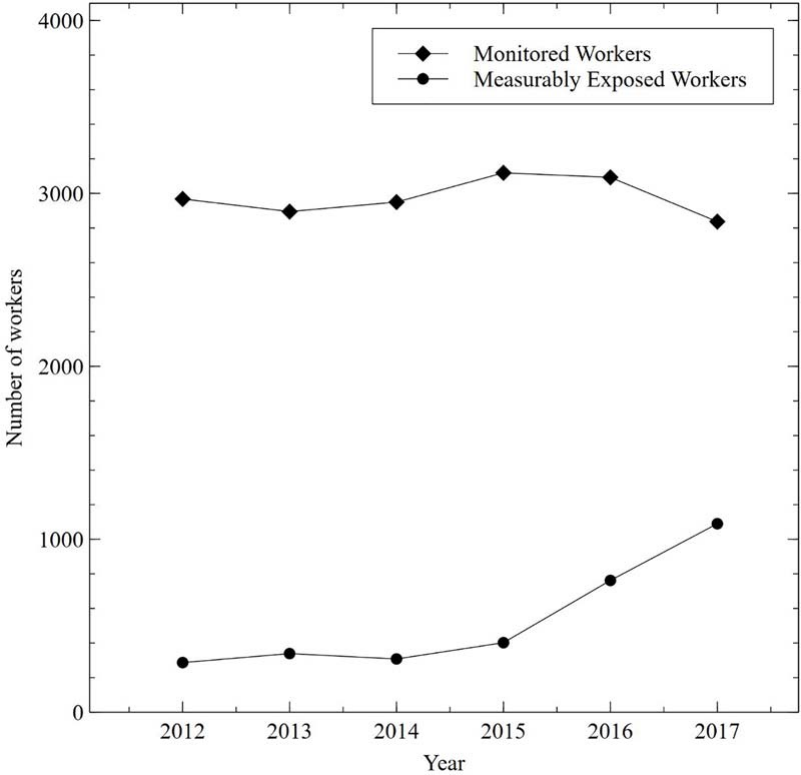
Comparison in the trend of the total number of monitored workers versus the number of measurably exposed workers.

**Figure 3:**
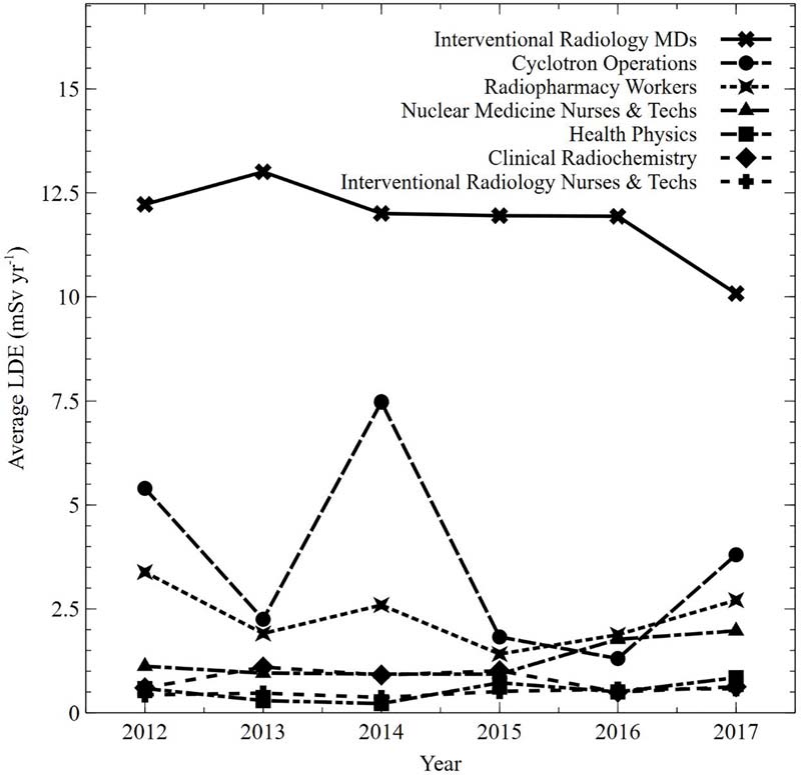
Average dose (mSv y^−1^) to the eye lens by medical staff category from 2012 to 2017 (average lens dose equivalent [LDE] for 2015 was interpolated for interventional radiology MDs based on 2014 and 2016 values).

**Figure 4:**
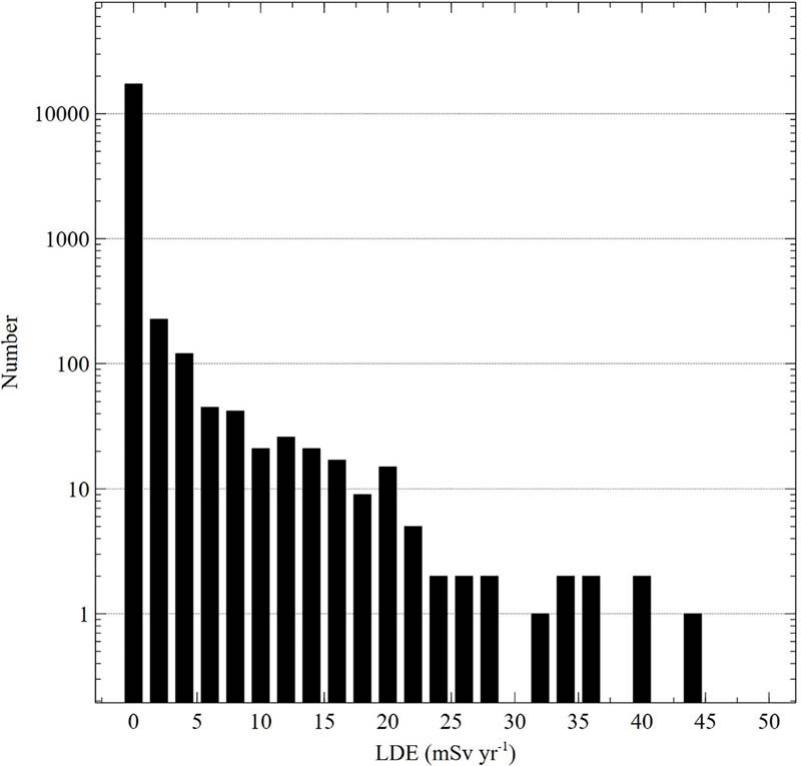
Distribution of annual occupational lens dose equivalent (LDE) measurements (mSv y^−1^) over 6 years at a high-volume medical institution.

Average, maximum and percentile radiation dose to the eye lens for staff categories are presented in Table 1. The averages are presented both for all monitored workers and the subset of monitored workers with measurable exposures. The average annual trends for all monitored workers versus measurably exposed workers are presented in Figure 1. Annual trends of the total number of monitored workers, and the number of measurably exposed workers, are presented in Figure 2. Categorized annual trends in average eye lens doses of medical staff are presented in Figure 3. One-year average distributions are presented in Figure 4, summarizing each measurable annual exposure for all workers.

The occupational dose to the eye lens for the entire hospital worker cohort rose by 103% over this study period, whereas the number of monitored workers remained relatively constant. Hence, there was an increase in average worker dose when considering all monitored workers as shown in Figure 1. However, when considering only measurably exposed workers, there was a 53% decline in the average dose. The increase in the number of positron emission tomography (PET) procedures, and IR/FGI procedures, was compensated by a larger increase in the workers assigned to those procedures and as the result of increased emphasis on as low as reasonably achievable (ALARA) work practices in these areas. The steep decline in the average dose of measurably exposed workers in the face of rising cumulative doses is explained by the strategic increase in the number of measurably exposed workers as shown in Figure 2.

FGI physicians (without considering protection from glasses) have an average of 11 mSv y^−1^_,_ the largest lens dose equivalent (LDE) of any group and have a maximum LDE of 45 mSv y^−1^, which is also the largest of any monitored individual. Cyclotron workers, radiopharmacy workers and nuclear medicine technicians/nurses have average doses of 4.4, 2.6 and 2.2 mSv, respectively. It is therefore important to optimize procedures and consider protective equipment for FGI physicians, cyclotron operators and workers with routine exposures to radiopharmaceuticals^(34)^. Inpatient nurses have the lowest average LDE of 0.02 mSv y^−1^, if all monitored nurses are considered. This average is 0.26 mSv y^−1^ when considering only nurses with measurable exposures.

Medical doctors and medical fellows conducting IR procedures and FGI have the largest average and maximum eye lens dose of any group in the hospital. The average dose to exposed members of this group was 11 mSv y^−1^ and as a group contributed 31% of the cumulative occupational dose to hospital workers. This group’s maximum lens dose in a single year was 45 mSv. Factors contributing to this worker’s large eye dose were extensive fluoroscopy use, correct and consistent OSL badge use and their relatively short height. In Figure 3, the average LDE for 2015 was interpolated for IR MDs based on 2014 and 2016 values due to insufficient data quality associated with the personnel monitoring process. The doses presented in this paper do not account for shielding from protective eyewear and are derived from measurements taken from an OSL personal dosemeter when on the user’s collar. The use of leaded glasses by this group reduced the actual doses to the lens^(9,10,20)^.

Workers who were exposed to radionuclides as a routine part of their occupational duties had significantly lower eye lens doses than fluoroscopy workers. For example, cyclotron support staff and radiopharmacy workers had average doses of 4.3 and 2.3 mSv y^−1^, respectively. Nuclear medicine nurses and technicians had an average dose of 1.3 mSv y^−1^. However, though the average eye dose is small, because of the large numbers of nurses, this group unsurprisingly^(36)^ contributes a substantial fraction (17%) of the total hospital eye lens dose. PET procedures are likely the primary contributor to collective dose from radiopharmaceutical exposure.

There are a limited set of published results with which direct comparison is possible. Dauer *et al*.^(19)^ reported unshielded operator LDEs obtained from dosimetry monitors worn outside the collar shield of operating IR in 2006 with a mean LDE and maximum LDE of 35.7 and 89.9 mSv, respectively. Betti *et al*.^(16)^ summarized eye lens doses of 15 cardiologists working in five operating rooms with an estimated mean and maximum annual LDE of 10 and 27.3 mSv, respectively. As shown in Table 1, average and maximum LDE values were substantially lower than both the Dauer *et al*. and Betti *et al*. studies with values of 11 and 45 mSv, respectively. A study conducted by Borrego *et al*.^(37)^, which considers workers who performed, or assisted with, FGI procedures reported a median LDE of 6.9 and 7.1 mSv, respectively, depending on the protocol.

Occupational LDE exposures among medical workers are known to be above average^(9)^. Radiation Exposure Information and Reporting System (REIRS) also provides some context of typical eye lens doses in the US worker population. The US NRC maintains the REIRS database and publishes annual reports based on radiation exposure records for each monitored NRC licensed individual. These reports are analyzed for trends and presented annually on the REIRS website^(38)^. Each annual report summarizes the maximum occupational doses for each exposure category, including LDE, the number of individuals with measurable dose and the number of individuals with LDE over 25% of the 0.15 Sv annual dose limit. According to the NRC annual reports from 2012 to 2017, the fraction of measurably exposed workers with LDE over 25% of the dose limit was 0.024%; the corresponding fraction for the MSK population was 0.089%. Significant differences exist between the populations summarized in the NRC annual reports and the MSK workers in the current report. The NRC annual report consists primarily of workers in commercial light-water reactors, industrial radiographers and fuel-cycle workers, with practically no monitored hospital workers. In addition, all the MSK workers with a reported LDE over 25% were IR physicians who routinely wore eye protection and therefore their actual doses would be significantly lower than reported.

No MSK worker had eye lens dose measurement that exceeded current US radiation dose limits. The largest recorded eye lens dose over the study period was 30% of the 150 mSv annual limit. The current ICRP lens of eye limit^(3)^ is not currently recognized in US regulatory code, but recommends a dose limit of 20 mSv averaged over 5 years with no single year above 50 mSv. In this work, no worker exceeded the 50 mSv recommendation of the ICRP, but there were several instances where the limit was approached, as shown in Figure 4. However, two IR physicians exceeded the ICRP lens of eye dose limit recommendation with 5-year averaged doses of 21 and 40 mSv, respectively.

MSK workers are carefully monitored, and exposures are maintained as low as reasonably allowable in accordance with regulatory guidelines. As discussed before, IR physicians as a group receive the largest dose to the eye lens due to the large number of fluoroscopy procedures conducted per year. These workers are carefully monitored, wear both a body and a collar badge, and are required by institutional policy to wear lead aprons and leaded eyewear during fluoroscopy procedures. Cyclotron operators employ remote manipulators when working with large activities of radionuclides. Leaded glass shields and shielded hoods are used when working with moderate-to-large activities of radionuclides. Several strategies have been implemented that reduce dose to nuclear medicine nurses and technicians. One effective strategy is to ensure that enough workers are available to conduct the PET injections, thus maintaining a low average by spreading out the cumulative dose; shielding solutions are also commonly employed. Inpatient nurses are protected by large rolling lead shields when caring for patients with large radiopharmaceutical burdens. Shielded syringes and automatic dose injectors have also been used to administer doses as an alternative to manual injection practices^(42)^. Radiation safety training is an important aspect of dose reduction. Workers frequently interact with the radiation protection staff with the goal of optimizing all exposures.

## LIMITATIONS

In this publication, annual doses have been presented for 5950 unique workers without in-depth analysis of the underlying monthly data. Thus, annual doses may in some cases be underestimated due to lost, unused or improperly worn badges. Lost-badge dose estimates were made only if an exposure was thought to have occurred during the lost monitoring period. In addition, improper positioning of personal dosemeters, such as switching collar and trunk dosemeters, may result in incorrectly calculated doses. Although these effects have not been quantified, they are expected to be small because workers are trained annually and typically wear their badges correctly. Several worker groups have a broad list of responsibilities and may not neatly fit in any single category. In such cases, workers were assigned to the category most closely aligned to their primary job responsibility.

## CONCLUSIONS

In this work, eye lens dose for workers in a high-volume oncologic medical institution have been presented. Data were collected over a period 2012–17 from OSL badge personal monitoring. A total of 5950 workers were included in the study. It is important to differentiate between monitored workers and the subset with measurable exposures (>0.1 mSv) because of the large difference in average and median doses for these two categories. In this study, the average eye lens dose for all monitored workers was 0.29 mSv y^−1^, whereas the average dose to measurably exposed workers was 1.6 mSv y^−1^. Nurses and technicians involved in PET injections received 17% of the center’s cumulative eye lens dose while maintaining a low average dose. Fluoroscopy workers had the highest cumulative, average and maximum doses of any group in the study, and monitoring is definitely required by regulation. However, the published dose for fluoroscopy workers represents a substantial overestimate because protection from utilized leaded glasses was not considered. Although none of the worker measurements exceed current US NRC dose limits, two workers exceeded the ICRP 5-year average dose limit recommendation.
